# Working Memory as the Focus of the Bilingual Effect in Executive Functions

**DOI:** 10.3390/bs15020134

**Published:** 2025-01-26

**Authors:** Jiejia Chen, Zitong Li, Zhiheng Xiong, Guangyuan Liu

**Affiliations:** 1 School of Electronic and Information Engineering, Southwest University, Chongqing 400715, China; chenlele1993@email.swu.edu.cn; 2 Key Laboratory of Cognition and Personality, Ministry of Education, Southwest University, Chongqing 400715, China; 3 School of Foreign Language, BeiHang University, Beijing 100191, China; zitong.li.22@ucl.ac.uk; 4 School of Humanities, Southeast University, Nanjing 211189, China; xzh_psy@seu.edu.cn

**Keywords:** bilingual effect, executive functions, visual and auditory modalities, inhibitory control, working memory, cognitive flexibility

## Abstract

The bilingual effect on executive functions (EFs) has garnered considerable attention, with most studies focusing on the visual domain and largely overlooking the auditory domain. Furthermore, research has predominantly concentrated on specific subcomponents of executive functions, with few studies systematically examining all three key subcomponents. This raises two important questions: (a) Is the bilingual effect specific to certain modalities (modality-specific), or a more general phenomenon (modality-general)? (b) Is the bilingual effect concentrated in a specific component of executive functions (process-specific), or does it extend to all three components (process-general)? To explore these questions, this study recruited monolingual Chinese and bilingual Chinese–English participants, using matched visual and auditory Stroop, N-back, and task-switching tasks to assess inhibitory control, working memory, and cognitive flexibility in both groups. The results showed that, after controlling for variables like intelligence, socioeconomic status, and age, bilingualism significantly predicted performance in both auditory and visual working memory tasks, explaining 34% and 19% of the variance, respectively. However, no evidence was found to support a bilingual effect in inhibitory control or cognitive flexibility. In conclusion, these results suggest that bilingual effects are not only process-specific (affecting only working memory) but also modality-general (providing advantages in both visual and auditory modalities).

## 1. Introduction

Executive functions (EFs) are a set of cognitive abilities that regulate people’s thoughts and behaviors (Chen et al., 2021; Diamond, 2013; Friedman & Miyake, 2017; Miyake et al., 2000). We rely on EFs to concentrate, switch between tasks, and suppress instinctive impulses in order to make rational choices. In other words, EFs are the cognitive foundation for managing daily life (Doebel, 2020; Nancy et al., 2008; Snyder, 2013). Individuals with weak EFs tend to have poor self-control and act impulsively, which can contribute to issues such as obesity, poor work performance, marital discord, and other self-management problems (Crescioni et al., 2011; Eakin et al., 2004). Since EFs are crucial for human development, improving them has become a widely discussed topic in psychology and neuroscience (Chen et al., 2020; Diamond & Ling, 2016; Frischen et al., 2021).

Learning a second language may be an effective way to improve executive functions (EFs) compared to other experiences, such as cognitive training with monotonous computer tasks. This cognitive advantage is referred to as the bilingual effect (Bialystok, 2017; Diamond, 2020; Thorell et al., 2009). The bilingual executive processing advantage hypothesis suggests that both language systems in the brain are activated when bilinguals process information. The brain then suppresses interference from the non-target language during this executive process, constantly monitoring, updating, and switching to the target language. This process likely involves a variety of cognitive functions, particularly EFs (Bialystok & Craik, 2022; Hannaway et al., 2019; Hilchey & Klein, 2011). Language use is one of the most intense, persistent, and integrated human experiences (Bialystok, 2017). Using language activates multiple brain regions, including the frontal, temporal, and parietal lobes (Bialystok & Craik, 2022; Friederici, 2011; Li et al., 2014). This extensive involvement of brain centers increases the likelihood that the experience-related effects of language use may generalize beyond language itself (Bialystok, 2017; Bialystok et al., 2012). Bilingual experience and EFs may recruit similar cognitive processes and brain networks, thereby facilitating the transfer of abilities between the two functions (Diamond & Ling, 2016; Miyake & Friedman, 2012).

However, research on the relationship between bilingual experience and executive functions (EFs) has produced inconsistent results, with some studies confirming a bilingual effect, while others have not (Anton et al., 2019; Blumenfeld & Marian, 2011; Keijzer, 2013; Privitera et al., 2022, 2023; Yang et al., 2018). EFs consist of three core components—inhibitory control, working memory, and cognitive flexibility (Diamond, 2013; Diamond & Ling, 2016). In terms of inhibitory control, Bialystok’s (Bialystok et al., 2014) study found that compared to monolinguals, bilinguals showed less interference in the visual Stroop task, suggesting an advantage in visual inhibitory control. However, recent studies have not found a significant difference in visual inhibitory control between bilinguals and monolinguals (Dick et al., 2019; Giguere et al., 2022). In terms of working memory, Anton et al. (2019) found that bilingual participants performed better in both visual and auditory reverse-order digit tasks compared to monolingual participants, suggesting that bilinguals have an advantage in both visual and auditory working memory. In contrast, other studies using the Working Memory Span Task revealed no visual working memory advantage for bilinguals (Keijzer, 2013; Ratiu & Azuma, 2014). For cognitive flexibility, the results of visual experiments are also controversial. Yang et al. (2018) found that bilinguals performed better in the Dimensional Change Card Sort task than monolinguals, indicating that bilinguals may possess stronger cognitive flexibility. However, Han et al.’s (2022) study found no relationship between visual cognitive flexibility and language switching ability.

To summarize, the inconsistent results of previous studies can be attributed to the following causes. First, although the auditory domain is more important in language acquisition and use than the visual domain, most studies have focused on the visual domain to examine the advantageous effects of bilingualism on executive functions (EFs). Few comparative studies on visual and auditory modalities exist. Most of this research has only examined the bilingual advantage in EFs within a single modality. Direct comparisons of the visual and auditory modalities are challenging due to differences in experimental paradigms, materials, procedures, and participants. As a result, the modality specificity or generality of this effect remains unclear. To our knowledge, no study has comprehensively examined the visual–auditory differences in the bilingual effect based on the three components of EFs (i.e., inhibitory control, working memory, and cognitive flexibility). Second, some studies did not appropriately control for additional variables that affect EFs, such as IQ and socioeconomic status (SES), which led to mixed effects from these extraneous variables in the bilingual effect (Antoniou, 2019; Paap et al., 2015; Xie & Pisano, 2019; Xie & Zhou, 2020). Specifically, IQ affects executive functions, with higher intelligence levels being associated with stronger executive functions (Xie & Pisano, 2019). Similarly, higher socioeconomic status (SES), which typically reflects better family conditions, is often linked to improved executive functions (Calvo & Bialystok, 2014). Third, studies used different criteria to select and assign participants as monolinguals or bilinguals (Bialystok et al., 2016; Zhou & Privitera, 2024). Moreover, most studies on bilingualism used self-report questionnaires to collect participants’ language information. However, the design and interpretation of such tools are vague and depend strongly on the validity of the participants’ subjective reports (Antoniou, 2019). These issues make cross-group comparisons difficult, and as a result, the relationship between bilingual experience and EFs remains unclear.

This study recruited bilingual and monolingual participants and investigated the relationship between executive functions (inhibitory control, working memory, and cognitive flexibility) and bilingual experience through auditory and visual tasks. The aim was to explore whether the bilingual effect on executive functions is modality-specific or modality-general, and whether it is process-specific or process-general. To address this, we matched the experimental procedures for both auditory and visual tasks to prevent potential interference arising from variations in the experimental design. Additionally, we incorporated a range of covariates, such as IQ, SES, and age, in our analysis to gain a more comprehensive understanding of the specific link between bilingual experience and executive functions. Furthermore, we used the outcomes of the International English Language Testing System, Class A (IELTS), and the College English Test Band 4 (CET-4, a national English proficiency test administered by China’s Higher Education Department) to measure participants’ proficiency in English as a second language (native Chinese). A language and social background questionnaire was also administered to assess the frequency of language use, which allowed for a more objective categorization of participants into bilinguals and monolinguals.

Based on this, this study systematically investigated the relationship between bilingual training and executive functions, focusing on the following two main research questions: Research Question a: Is the bilingual effect specific to certain modalities (modality-specific), or a more general phenomenon (modality-general)? If bilinguals outperform in either auditory or visual tasks, the bilingual effect on executive functions would be considered modality-specific. Conversely, if they outperform in both auditory and visual tasks, this would indicate a modality-general effect. Research Question b: Is the bilingual effect concentrated in a specific component of executive functions (process-specific), or does it extend to all three components (process-general)? If bilinguals demonstrate superiority in only one core component of executive functions (e.g., working memory) without a corresponding advantage in the other components, the bilingual effect would be process-specific. However, if the bilingual effect is observed across all three components, it would suggest a process-general effect.

## 2. Materials and Methods

### 2.1. Participants

In this experiment, 60 participants were divided into two groups: 30 bilinguals and 30 monolinguals. The bilinguals had a total IELTS score of 7.0 or above, indicating a good proficiency in English and the ability to communicate effectively in English, which improved the grouping validity. In contrast, all participants in the monolingual group failed the CET-4 test, scoring less than 350 out of 710, indicating a poor grasp of English and an inability to communicate in English.

All participants completed the simplified version of the Raven’s Advanced Intelligence Test (Arthur & Day, 1994) (12 questions, 1 point per question, with a total score of 12) and the Language and Social Background Questionnaire (Chinese version) (Anderson et al., 2018) to collect information about their bilingual experience (Table 1). Social background information included participants’ names, gender, age, and SES. Specifically, participants’ names were recorded solely for the purpose of matching their survey and behavioral experiment results. In accordance with privacy and confidentiality principles, all personal information was anonymized during data analysis. Gender was recorded as a categorical variable, with 0 for male and 1 for female. Age was reported by participants in years, rounded to the nearest integer. SES was measured by the highest level of education of their parents, scored on a 1–5 scale: “Elementary school or below” = 1; “Junior high school” = 2; “High school or vocational school” = 3; “University” = 4; “Postgraduate or above” = 5 (Chen et al., 2020).

All participants were right-handed, had no history of psychiatric or neurological illness, and were not taking medication at the time of the test. Participant data exceeding ± 3 standard deviations were excluded from the statistical analysis. This study was approved by the Ethics Committee of Southwest University, and the participants signed an informed consent form prior to the experiment onset and received a predetermined reward upon completion. After excluding four participants (one who failed to complete the experiment as required, and three whose results exceeded 3 standard deviations), twenty-eight participants from each group were included in the statistical analysis. Both groups were matched by age (*t*_(54)_ = 0.65, *p* > 0.2), gender ($\chi^{2}$_(1)_ = 0.13, *p* > 0.2), SES (*t*_(54)_ = 0.51, *p* > 0.05), and IQ (*t*_(54)_ = 1.45, *p* > 0.05).

### 2.2. Materials

An experienced researcher conducted the experiment in a quiet laboratory and provided one-on-one guidance to the participants. Six well-known experimental paradigms were used to measure the three main components of executive functions (EFs) through visual and auditory channels: the visual Stroop test, auditory Stroop test, visual N-back task, auditory N-back task, visual task-switching test, and auditory task-switching test. The experiment was divided into three parts, with two tasks completed at a time (each lasting about 10–15 min), and a 15 to 20 min break between each part to prevent participant fatigue. The order of the six tasks was counterbalanced across participants. The participants completed the demographic information, language questionnaires, and intelligence tests after finishing the experimental tasks. The total duration of the experiment was approximately 2 to 2.5 h, including rest time.

#### 2.2.1. Visual Stroop Task

The materials in the visual Stroop test consisted of 16 Chinese characters, which included the words “red”, “green”, “blue”, and “yellow” in four different colors. Stimuli were divided into word–color-consistent trials (congruent) and word–color-inconsistent trials (incongruent). Participants made judgments about the color of the word and ignored its meaning. In total, 160 trials were conducted, with 80 trials each for congruent and incongruent conditions. The instructions were presented before the experiment began. First, a black “+” appeared in the center of the screen for 500 ms. Next, a random blank screen flashed for 500–800 ms, and a stimulus appeared for 300 ms. The stimulus was followed by a blank response screen for 1000 ms and a random blank screen for 1000–1500 ms. The differences in accuracy and reaction times between the congruent and incongruent conditions represent the Stroop interference effect. A smaller effect indicates better inhibitory control in response to visual stimuli.

#### 2.2.2. Auditory Stroop Task

The materials in the auditory Stroop test included eight pronunciations of the Chinese characters “big” and “small” at different volumes (70 dB/50 dB) in male or female voices. Based on whether the loudness matched the meaning, they were divided into consistent (congruent: 70 dB for “big” and 50 dB for “small”) and inconsistent trials (incongruent: 70 dB for “small” and 50 dB for “big”). The experimental process was the same as the visual Stroop test, and the presentation time for the sound stimulus was 300 ms. Before the main experiment, 30 college students with normal hearing were invited to evaluate the sound materials on a scale of 1–5. The results showed that all the sounds were clear and varied (*M* = 4.86, *SD* = 0.35), with neutral emotions (*M* = 4.80, *SD* = 0.41), and there was a significant difference in perceived volume between the two levels (*M* = 4.93, *SD* = 0.25).

#### 2.2.3. Visual N-Back Task

The visual N-back task consisted of three blocks: 1-back, 2-back, and 3-back, presented in sequence. Each block included a practice session, followed by the formal experiment. A total of 180 trials were conducted, with 60 trials for each block. The procedure for each trial was as follows: the experimental instructions were presented at the start of the test, followed by a blank screen for 1000 ms, a visual stimulus for 300 ms, a black response screen for 1500 ms, and a random blank screen that lasted between 1000 ms and 1500 ms. Task accuracy (proportion of hits minus proportion of false alarms) under different working memory loads was used as the evaluation metric. Higher accuracy and shorter response times indicated stronger working memory performance under visual stimulation.

#### 2.2.4. Auditory N-Back Task

The materials for the auditory N-back task consisted of the numbers 1–9 spoken in both male and female voices. The experimental procedure was the same as that of the visual N-back task, with a total of 180 trials, 60 trials in each block. All sound stimuli were clear, identifiable, and emotionally neutral, assessed using the same procedure as the auditory Stroop task.

#### 2.2.5. Visual Task-Switching Paradigm

The materials for the visual task-switching paradigm consisted of visual presentations of the numbers 1–9. Participants were asked to respond to cues in two types of tasks. The ‘size’ cue task required participants to judge whether a number was greater than 5, pressing the F key for numbers less than 5 and the J key for numbers greater than 5. The number 5 did not appear in this task. The ‘parity’ cue task required participants to judge whether a number was odd or even, pressing the F key for odd numbers and the J key for even ones. The formal experiment consisted of two blocks, with a total of 122 mixed trials. The task requirements were switched randomly between trials, categorizing them into either switch (60 trials) or stay conditions (60 trials). A stay trial was when the task requirement was the same as the previous trial, whereas a switch trial differed from the previous trial. Each trial began with a 500 ms fixation point (“+”), followed by a 200 ms cue stimulus (“size” or “parity”) to remind participants of the current task, then a random blank screen for 300–500 ms. The visual stimulus was presented for 300 ms, followed by a blank response screen for 1500 ms, a random blank screen for 1000–1200 ms, and the next trial. Switch costs were the difference in accuracy between stay and switch trials. Smaller switch costs indicated better switching performance in response to visual stimulation.

#### 2.2.6. Auditory Task-Switching Paradigm

The materials for the auditory task-switching paradigm consisted of the numbers 1–9 spoken in male and female voices. Each auditory stimulus lasted 300 ms and was clear, identifiable, and emotionally neutral. The experimental process was the same as the visual task-switching paradigm.

## 3. Results

SPSS Version 24.0 was used for the statistical analysis. This study systematically examined the relationship between bilingual experience and executive functions using three main methods: independent samples *t*-tests, correlation analysis, and multiple regression analysis. These methods aimed to address two key research questions: (a) Is the bilingual effect modality-specific or modality-general? (b) Is the bilingual effect process-specific or process-general?

First, to assess group differences, we conducted analysis of variance (ANOVA). For example, in the Stroop task, response time was analyzed using a two-factor variance ANOVA (2 × 2: conditions: congruent vs. inconsistent, participant type: bilingual vs. monolingual). Similarly, variance analysis was applied to the N-back task (3 × 2: conditions: 1-back, 2-back, 3-back, participant type: bilingual vs. monolingual) and the task-switching paradigm (2 × 2: conditions: stay vs. switch, participant type: bilingual vs. monolingual). The *p*-values that did not meet the sphericity assumption were corrected using the Greenhouse–Geisser method. The results of *t*-tests were corrected by the false discovery rate method. However, due to space constraints and the fact that the ANOVA results were not central to our research questions, we chose not to present them in detail. Instead, we focused on reporting the independent sample *t*-test results, which are the core findings of this study.

Next, correlation analysis was conducted to examine the relationships between bilingual experience and executive functions. Finally, multiple regression analysis was performed to evaluate the predictive power of bilingual experience on executive functions, controlling for potential confounding variables such as age, socioeconomic status (SES), and IQ.

### 3.1. Differences in EFs Between Bilingual and Monolingual Groups

Independent sample *t*-tests were used to compare bilingual and monolingual participants on the EF tasks (see Table 2). In terms of inhibitory control, no significant differences were found between the two groups on the visual Stroop task (*ts*_(54)_ < 1.50, *ps* > 0.05). However, in the auditory domain, the bilingual group exhibited a significantly smaller Stroop effect compared to the monolingual group.

**Figure 1:**
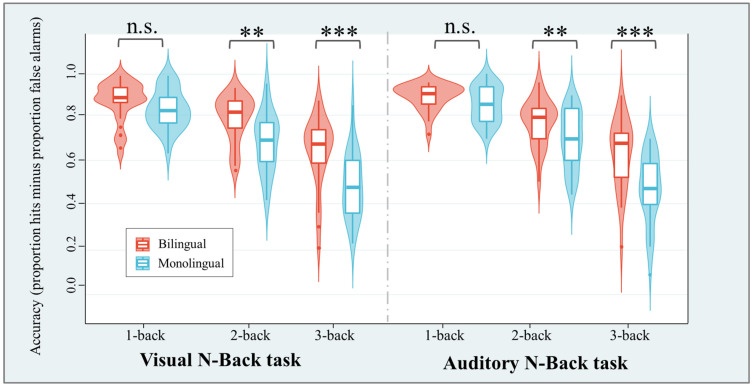
Accuracy rate and response time for bilinguals and monolinguals in the visual N-back task (**left**) and the auditory N-back task (**right**). ** *p* < 0.01; *** *p* < 0.001; n.s. not significant.

In terms of working memory, the bilingual group demonstrated significantly higher accuracy (proportion of hits minus proportion of false alarms) on both the visual and auditory tasks in the 2-back and 3-back conditions compared to the monolingual group (see Figure 1). However, the performance of the two groups was similar in the 1-back condition. These findings suggest that bilingual participants may possess superior working memory abilities in both the auditory and visual modalities.

Finally, in terms of cognitive flexibility, no significant differences were found between the two groups in terms of switch costs for both the auditory and visual task-switching paradigms (*ts*_(54)_ < 1.50, *ps* > 0.05). These findings suggest that there is no evidence to support the existence of a bilingual effect in cognitive flexibility, either in the visual or auditory modality.

### 3.2. Correlation Analysis: Bilingualism and Executive Functions

**Figure 2:**
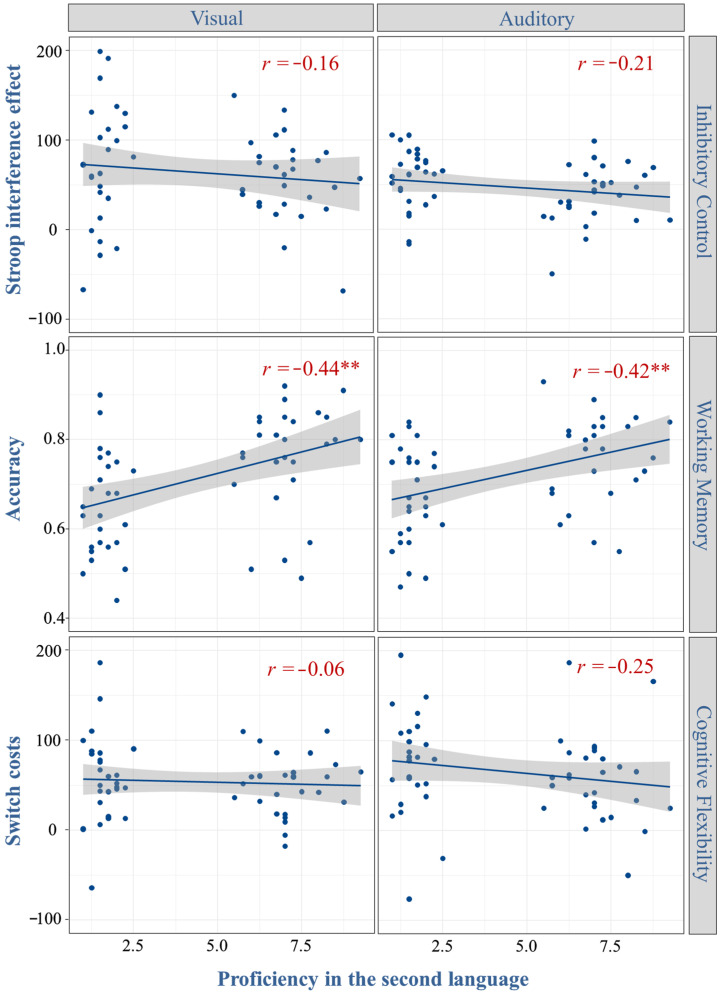
Scatter plots with correlation analysis between the proficiency in the second language and performances in six EF tasks. Stroop interference effect (Stroop task): incongruent minus congruent RTs. Accuracy (N-back task): proportion hits minus proportion false alarms). Switch costs (task-switch paradigms): switch minus stay RTs. ** *p* < 0.01.

The Pearson correlations presented in Figure 2 reveal potential associations between EF measures and bilingualism, suggesting that language proficiency may indeed be related to certain aspects of EF. Specifically, the results indicate that higher proficiency in the second language (L2) is predictive of improved performance on both auditory and visual working memory updating tasks. However, language proficiency does not appear to be linked with inhibitory control or cognitive flexibility. These findings suggest that the bilingual effect is more likely to manifest in the domain of working memory rather than in other cognitive abilities.

### 3.3. Multiple Regression Analyses

Both the group differences and correlation analyses did not account for potentially confounding variables that may influence EFs, such as age (Doebel, 2020), socioeconomic status (Hackman & Farah, 2009), and IQ (Hargreaves & Aksentijevic, 2011). To determine whether bilingualism can explain differences in EF task performance between the two groups, we conducted a multivariate multiple regression analysis on the six dependent measures (i.e., the six EF tasks: auditory and visual versions of inhibitory control, working memory, and cognitive flexibility), while accounting for the effects of age, socioeconomic status, and IQ. The results of the regression analysis were consistent with the correlation findings (see Table 3). Specifically, bilingualism can predict performance in both auditory and visual working memory tasks. However, it is not predictive of inhibitory control or cognitive flexibility abilities in either auditory or visual tasks.

## 4. Discussion

This study systematically explored the relationship between bilingual experience and executive functions (EFs) through matched visual and auditory tasks, aiming to determine whether the bilingual effect is modality-specific or modality-general (Research Question a), and whether it is process-specific or process-general (Research Question b). The correlation results showed a significant relationship between second-language experience and working memory, but no significant relationship was observed with inhibitory control or cognitive flexibility. Similarly, the regression analysis indicated that, after controlling for confounding variables such as intelligence, socioeconomic status, and age, bilingualism predicted performance in both auditory and visual working memory tasks. However, no bilingual advantage was found in inhibitory control or cognitive flexibility.

These findings addressed our first research question by demonstrating that the bilingual effect was modality-general, as it was observed in both auditory and visual working memory tasks. In response to our second research question, the results indicated that the bilingual effect was process-specific, as it was limited to working memory and did not extend to inhibitory control or cognitive flexibility. Thus, our study provided evidence that bilingualism can enhance working memory, but this effect is specific to the process of working memory and applies across modalities (auditory and visual). The following discusses this result from two perspectives.

### 4.1. Bilingual Experience Predicts Working Memory

Specifically, our research findings indicate that bilingual experience can predict performance in visual and auditory working memory tasks, highlighting advantages for bilingual individuals in both visual and auditory working memory. Previous studies have also reported similar findings (Alain et al., 2018; Dong & Liu, 2016; Monnier et al., 2021). As an illustration, Dong and Liu (2016) conducted a longitudinal study demonstrating a noteworthy enhancement in the visual working memory capability of college students following a semester of interpretation and translation training. Additionally, a comprehensive meta-analysis conducted by Monnier et al. (2021), incorporating findings from 116 independent studies, indicated a modest effect size of 0.12, favoring a higher working memory capacity for bilingual individuals compared to monolinguals.

This result suggests that the dual language experience might have strengthened the working memory capacity of bilinguals (Alain et al., 2018; Antoniou, 2019; Bialystok, 2017). Many cognitive psychologists argue that executive functions, particularly working memory, are not isolated modules, but rather domain-general capacities closely linked to individual experiences (MacDonald & Christiansen, 2002; Schwering & MacDonald, 2020). Therefore, the potential of bilingual experience to enhance working memory can be attributed to its unique intervention characteristics. Previous research has identified several key features for improving working memory: Characteristic (1): Only challenging and progressively difficult training tasks can enhance an individual’s working memory; repetitive or simple task repetition does not contribute to improving working memory (Diamond, 2016; Diamond & Lee, 2011). Characteristic (2): Persistent and consistent training is crucial; the improvement of working memory significantly depends on the duration of the training (Diamond & Lee, 2011; Thorell et al., 2009). It is evident that bilingual experience meets both of these key characteristics. The Bilingual Interactive Activation (BIA) model, proposed by Dijkstra and Van Heuven, posits that a key characteristic of bilingual cognition is the simultaneous activation of both languages (Bialystok, 2017; Bialystok et al., 2016; Dijkstra & Van Heuven, 2002). When bilinguals encounter a stimulus, such as hearing or seeing a word, the lexical representations of both languages are activated, regardless of which language the stimulus originates from (Dijkstra & Van Heuven, 2013; Dijkstra & Van Heuven, 2002). This activation is automatic and non-selective, meaning it occurs without the individual’s intention or conscious control (Dijkstra & Van Heuven, 2013; Dijkstra & Van Heuven, 2002). In order to successfully identify and use the correct language, bilinguals must manage the ongoing competition between both languages. This process undoubtedly demands significant working memory resources. This demand is particularly pronounced in the context of interpreting, where simultaneous interpreters must rapidly convey the speaker’s message while simultaneously processing incoming information (Antoniou, 2019; Bialystok et al., 2012; Diamond, 2013). Secondly, the learning of a second language inherently involves a progression from easy to difficult, following a cyclical and progressively challenging pattern that requires long-term commitment. Therefore, it is reasonable to assume that this training would enhance the working memory of bilingual individuals.

From a neuroscience perspective, the enhancement of working memory in bilinguals may stem from changes in the function and structure of brain regions involved in working memory. Functionally, Alain et al. (2018) utilized functional magnetic resonance imaging (fMRI) in conjunction with the N-back task and found that bilinguals exhibited an advantage in auditory working memory. Specifically, bilinguals showed lower activation in the prefrontal cortex compared to monolinguals, suggesting that bilinguals use neural resources more efficiently, thereby successfully engaging in the working memory task (Alain et al., 2018; Huang et al., 2013). Furthermore, bilinguals may exhibit greater functional connectivity within and across networks relevant to executive control, highlighting the more flexible and efficient coordination between different neural regions and networks facilitated by bilingual experience (Grady et al., 2015). Structurally, bilingual experience has been found to increase gray matter density in regions implicated in executive control, such as the dorsolateral prefrontal cortex (DLPFC), left caudate nucleus (LCN), and anterior cingulate cortex (ACC) (Hayakawa & Marian, 2019; Olulade et al., 2016). These brain regions and networks are crucial for supporting working memory. Therefore, the transfer effects of bilingual experience on working memory may result from changes in the function and structure of these key brain regions. These beneficial changes, in turn, can enhance language learning and other cognitive functions (Hayakawa & Marian, 2019).

Some studies, however, have not observed a working memory advantage in bilinguals (Keijzer, 2013; Ratiu & Azuma, 2014), which may be attributed to the relatively lower difficulty levels of their tasks. According to the characteristics of working memory training research, the training effects on working memory tend to manifest in tasks with moderate difficulty levels, as overly simplistic or overly challenging tasks can lead to ceiling or floor effects, thereby diminishing the training effects on working memory (Diamond, 2013; Diamond et al., 2007). For example, a study employing a straightforward task, with reaction times typically less than 500 ms, did not find evidence of a bilingual effect (Bialystok et al., 2014). This may be attributed to the task’s simplicity, leading to a lack of sufficient sensitivity (Antoniou, 2019; Diamond, 2013). In contrast, bilinguals are more prone to showcase an advantage in tasks of greater complexity. Ma et al. (2020) demonstrated that the advantageous effect of proficient bilinguals was more prominent under conditions of high memory load. Our study also yielded similar results, revealing that bilinguals demonstrated significantly higher accuracy than monolinguals in the 2-back and 3-back conditions. However, no significant difference between the two groups was observed in the 1-back condition, and this effect remained consistent in both visual and auditory tasks. In summary, we posit that under conditions of high memory load, a clear relationship between bilingual experience and working memory capacity becomes evident.

### 4.2. Weak Relationship Between Bilingual Experience and Inhibitory Control and Cognitive Flexibility

We found that proficiency in a second language was not significantly related to inhibitory control and cognitive flexibility. Moreover, after accounting for relevant confounding variables such as intelligence, socioeconomic status, and age, bilingual experience did not predict performance in auditory and visual inhibitory control and cognitive flexibility tasks. This may suggest that bilinguals do not exhibit advantages in inhibitory control and cognitive flexibility in both visual and auditory modalities. Previous research has also uncovered similar results (Dick et al., 2019; Hilchey & Klein, 2011; Lehtonen et al., 2018). For instance, recent studies have failed to identify a significant difference in visual inhibitory control between bilinguals and monolinguals, indicating the absence of an inhibitory control advantage in bilinguals (Dick et al., 2019; Giguere et al., 2022). Similarly, in a study utilizing event-related potentials (ERPs) to investigate cognitive flexibility, no significant differences were observed between monolinguals and bilinguals in the core ERP components N2 and P3b, indicating the absence of a cognitive flexibility advantage in bilinguals (Lopez Zunini et al., 2019).

Previous research has reported advantages for bilinguals in inhibitory control or cognitive flexibility; however, there may not have been sufficient control for confounding variables related to EFs (Paap et al., 2015). That is, this “bilingual effect” can be attributed to the interference of additional variables. For example, Morton and Harper (2007) (conducted a study using the same task, methodology, and design as Bialystok et al. (2004). However, Morton’s study accounted for the socioeconomic status (SES) balance between bilinguals and monolinguals, while Bialystok’s study did not control for SES (Bialystok et al., 2004; Morton & Harper, 2007). Interestingly, Bialystok reported a bilingual effect, while Morton found a weaker relationship between bilingual experience and inhibitory control (Bialystok et al., 2004; Morton & Harper, 2007). Similarly, a meta-analytics study revealed a minimal bilingual effect for cognitive flexibility, and this advantage disappeared after correcting for bias (Lehtonen et al., 2018). Our study also underscores the importance of controlling for additional variables related to EFs. Initially, we observed a reduced Stroop effect in bilinguals, suggesting a potential advantage in inhibitory control. However, this advantage disappeared once we accounted for additional variables such as age, IQ, and SES. These findings suggest that extra variables associated with EFs may contribute to the observation of a false “bilingual effect”, emphasizing the need for comprehensive control of these variables in experimental settings.

### 4.3. Limitation

This study has several limitations that should be considered. First, a cross-sectional design was employed, comparing bilinguals and monolinguals, which cannot establish a causal relationship between bilingual experience and executive functions (EFs) and does not fully control for inherent individual differences between the two groups. To address this, we controlled for additional variables influencing EFs, including age, intelligence, and socioeconomic status (SES). Future research could use a longitudinal design to investigate causal links between bilingual experiences and EFs in different sensory modalities. Second, some studies contradict our findings, possibly due to factors such as participants’ age and specific bilingual experiences. Therefore, future research should carefully consider these factors (Degirmenci et al., 2022; Dong & Liu, 2016; Goodrich et al., 2022). Third, this study explored the bilingual effect only at the behavioral level. Future research could utilize techniques such as ERP or fMRI to explore the cognitive neural mechanisms underlying the bilingual effect on EFs, especially in relation to differences between visual and auditory modalities.

## 5. Conclusions

Most research on bilingual experience and executive functions has focused primarily on the visual domain, leaving uncertainty regarding whether bilingual effects differ across visual and auditory channels. To fill this gap, the present study systematically explored the relationship between bilingual experience and executive functions, specifically examining three subcomponents: inhibitory control, working memory, and cognitive flexibility. We recruited both monolingual Chinese and bilingual Chinese–English participants and designed controlled visual and auditory tasks to assess their executive function performance. This study aimed to answer two key research questions: (a) Is the bilingual effect modality-specific or modality-general? (b) Is the bilingual effect process-specific or process-general? The results showed that, after controlling for variables like intelligence, socioeconomic status, and age, bilingualism significantly predicted performance on both auditory and visual working memory tasks, explaining 34% and 19% of the variance, respectively. However, no evidence was found to support the presence of a bilingual advantage in inhibitory control or cognitive flexibility. In conclusion, the bilingual effect observed in this study was both process-specific and modality-general: it enhanced working memory performance but did not extend to inhibitory control or cognitive flexibility, being evident in both auditory and visual modalities. These findings contribute to a deeper understanding of the bilingual effect on executive functions, offering valuable insights for the design of foreign language education and further research in cognitive development.

## Figures and Tables

**Table 1:** Demographic and linguistic information of the Chinese monolingual and Chinese–English bilingual participants.

	Bilinguals *M* (*SD*)	Monolinguals *M* (*SD*)
Age	20.21 (1.34)	19.96 (1.58)
Gender	4 males, 24 females	5 males, 23 females
SES	3.23 (0.40)	3.18 (0.39)
Intelligence (out of 12)	9.68 (1.66)	9.36 (1.37)
L2 AoA	5.61 (1.66)	7.04 (0.64)
IELTS score (total)	7.21 (0.36)	--
IELTS score (understanding)	7.81 (0.75)	--
IELTS score (speaking)	6.45 (0.48)	--
IELTS score (reading)	7.71 (0.64)	--
IELTS score (writing)	6.50 (0.45)	--
Self-reported L2 understanding proficiency (none: 1–fluent (native): 10)	7.50 (1.30)	1.50 (0.69)
Self-reported L2 speaking proficiency (none: 1–fluent (native): 10)	6.61 (0.96)	1.43 (0.79)
Self-reported L2 reading proficiency (none:1–fluent (native): 10)	7.43 (1.29)	1.82 (0.82)
Self-reported L2 writing proficiency (none: 1–fluent (native): 10)	6.79 (1.13)	1.68 (0.86)
L2 use at home (All L1: 0%–All L2: 100%)	16.07% (20.65%)	--
L2 use in non-home situations (school) (All L1: 0%–All L2: 100%)	41.07% (18.28%)	--

Notes: SES: socioeconomic status; AoA: age of acquisition; L2: second language (English); and self-rated proficiency rated on a 10-point scale, where 1 is no ability at all, and 10 is fluent (native) ability. Language use was rated on a 5-point scale: 0%, 25%, 50%, 75%, and 100% of the time. Age of acquisition was reported for monolingual speakers because many received English instruction at school but never achieved fluency.

**Table 2:** Mean scores (M) and standard deviations (SD) of EFs measures in bilinguals and monolinguals.

EFs Measures	Bilinguals *M* (*SD*)	Monolinguals *M* (*SD*)	*p*-Values
Inhibitory Control (response time in ms)			
Visual Stroop			
Congruent	646 (72)	614 (76)	0.22
Incongruent	703 (75)	689 (89)	0.53
Difference (Stroop effect)	58 (45)	76 (71)	0.26
Auditory Stroop			
Congruent	610 (57)	639 (75)	0.10
Incongruent	648 (66)	696 (86)	0.03
Difference (Stroop effect)	39 (32)	56 (33)	0.05
Working Memory (accuracy: proportion of hits minus proportion of false alarms)			
Visual N-back			
1-back	0.88 (0.09)	0.84 (0.09)	0.10
2-back	0.78 (0.11)	0.67 (0.14)	0.001
3-back	0.63 (0.16)	0.47 (0.15)	<0.001
Combined	0.76 (0.12)	0.66 (0.11)	0.001
Auditory N-back			
1-back	0.89 (0.06)	0.86 (0.08)	0.06
2-back	0.78 (0.11)	0.69 (0.13)	0.01
3-back	0.63 (0.16)	0.47 (0.16)	<0.001
Combined	0.77 (0.10)	0.67 (0.11)	0.001
Cognitive Flexibility (response time in ms)			
Visual Task-switching			
Stay	813 (118)	824 (109)	0.71
Switch	864 (122)	881 (112)	0.71
Difference (Switch costs)	51 (32)	56 (49)	0.62
Auditory Task-switching			
Stay	861 (98)	952 (79)	<0.001
Switch	919 (128)	1036 (79)	<0.001
Difference (Switch costs)	57 (48)	84 (66)	0.09

**Table 3:** Summary of univariate regression analyses.

Predictors	Inhibitory Control (incongruent minus congruent RTs)			
	Visual		Auditory	
	*β*	*t*	*β*	*t*
Age	−0.14	−0.83	0.21	1.34
SES	0.25	1.77	−0.06	−0.42
IQ	0.02	0.11	−0.19	−1.28
Bilingualism	−0.16	−1.19	−0.26	−1.94
$R^{2}$/*adj.*$R^{2}$	0.08/0.01		0.11/0.04	
Predictors	Working Memory (proportion hits minus proportion false alarms)			
	Visual		Auditory	
	*β*	*t*	*β*	*t*
Age	0.11	0.84	−0.02	−0.16
SES	−0.04	−0.31	0.08	0.62
IQ	0.42	3.32 **	0.24	1.77
Bilingualism	0.37	3.35 **	0.39	3.20 **
$R^{2}$/*adj.*$R^{2}$	0.39/0.34		0.25/0.19	
Predictors	Cognitive Flexibility (switch minus stay RTs)			
	Visual		Auditory	
	*β*	*t*	*β*	*t*
Age	−0.13	−0.82	0.03	0.22
SES	−0.15	−1.06	−0.05	−0.30
IQ	0.02	0.13	−0.11	−0.75
Bilingualism	−0.05	−0.35	−0.22	−1.60
$R^{2}$/*adj.*$R^{2}$	0.06/−0.02		0.07/−0.01	

Bilingualism: 1 = bilingual participants; −1 = monolingual participants. ** *p* < 0.01.

## Data Availability

The data presented in this study are available on request from the corresponding author (the data are not publicly available due to privacy or ethical restrictions).

## References

[B1-behavsci-15-00134] Alain C., Khatamian Y., He Y., Lee Y., Moreno S., Leung A. W. S., Bialystok E. (2018). Different neural activities support auditory working memory in musicians and bilinguals. Annals of the New York Academy of Sciences.

[B2-behavsci-15-00134] Anderson J. A. E., Mak L., Chahi A. K., Bialystok E. (2018). The language and social background questionnaire: Assessing degree of bilingualism in a diverse population. Behavior Research Methods.

[B3-behavsci-15-00134] Anton E., Carreiras M., Dunabeitia J. A. (2019). The impact of bilingualism on executive functions and working memory in young adults. PLoS ONE.

[B4-behavsci-15-00134] Antoniou M. (2019). The advantages of bilingualism debate. Annual Review of Linguistics.

[B5-behavsci-15-00134] Arthur W., Day D. V. (1994). Development of a short-form for the raven advanced progressive matrices test. Educational and Psychological Measurement.

[B6-behavsci-15-00134] Bialystok E. (2017). The bilingual adaptation: How minds accommodate experience. Psychological Bulletin.

[B7-behavsci-15-00134] Bialystok E., Abutalebi J., Bak T. H., Burke D. M., Kroll J. F. (2016). Aging in two languages: Implications for public health. Ageing Research Reviews.

[B8-behavsci-15-00134] Bialystok E., Craik F. I. M. (2022). How does bilingualism modify cognitive function? Attention to the mechanism. Psychonomic Bulletin & Review.

[B10-behavsci-15-00134] Bialystok E., Craik F. I. M., Klein R., Viswanathan M. (2004). Bilingualism, aging, and cognitive control: Evidence from the simon task. Psychology and Aging.

[B9-behavsci-15-00134] Bialystok E., Craik F. I. M., Luk G. (2012). Bilingualism: Consequences for mind and brain. Trends in Cognitive Sciences.

[B11-behavsci-15-00134] Bialystok E., Poarch G., Luo L., Craik F. I. M. (2014). Effects of bilingualism and aging on executive function and working memory. Psychol Aging.

[B12-behavsci-15-00134] Blumenfeld H. K., Marian V. (2011). Bilingualism influences inhibitory control in auditory comprehension. Cognition.

[B13-behavsci-15-00134] Calvo A., Bialystok E. (2014). Independent effects of bilingualism and socioeconomic status on language ability and executive functioning. Cognition.

[B14-behavsci-15-00134] Chen J., Scheller M., Wu C., Hu B., Peng R., Liu C., Liu S., Zhu L., Chen J. (2021). The relationship between early musical training and executive functions: Validation of effects of the sensitive period. Psychology of Music.

[B15-behavsci-15-00134] Chen J., Zhou Y., Chen J. (2020). The relationship between musical training and inhibitory control: An ERPs study. Acta Psychologica Sinica.

[B16-behavsci-15-00134] Crescioni A. W., Ehrlinger J., Alquist J. L., Conlon K. E., Baumeister R. F., Schatschneider C., Dutton G. R. (2011). High trait self-control predicts positive health behaviors and success in weight loss. Journal of Health Psychology.

[B17-behavsci-15-00134] Degirmenci M. G., Grossmann J. A., Meyer P., Teichmann B. (2022). The role of bilingualism in executive functions in healthy older adults: A systematic review. International Journal of Bilingualism.

[B18-behavsci-15-00134] Diamond A. (2013). Executive functions. Annual Review of Psychology.

[B19-behavsci-15-00134] Diamond A. (2016). Why improving and assessing executive functions early in life is critical. Executive function in preschool age children: Integrating measurement, neurodevelopment and translational research.

[B20-behavsci-15-00134] Diamond A. (2020). Executive functions. Handbook of Clinical Neurology.

[B21-behavsci-15-00134] Diamond A., Barnett W. S., Thomas J., Munro S. (2007). The early years—Preschool program improves cognitive control. Science.

[B22-behavsci-15-00134] Diamond A., Lee K. (2011). Interventions shown to aid executive function development in children 4 to 12 years old. Science.

[B23-behavsci-15-00134] Diamond A., Ling D. S. (2016). Conclusions about interventions, programs, and approaches for improving executive functions that appear justified and those that, despite much hype, do not. Developmental Cognitive Neuroscience.

[B24-behavsci-15-00134] Dick A. S., Garcia N. L., Pruden S. M., Thompson W. K., Hawes S. W., Sutherland M. T., Riedel M. C., Laird A. R., Gonzalez R. (2019). No evidence for a bilingual executive function advantage in the nationally representative ABCD study. Nature Human Behaviour.

[B25-behavsci-15-00134] Dijkstra T., Van Heuven W. J. B. (2002). The architecture of the bilingual word recognition system: From identification to decision. Bilingualism-Language and Cognition.

[B26-behavsci-15-00134] Dijkstra T., Van Heuven W. J. B. (2013). The BIA model and bilingual word recognition. Localist connectionist approaches to human cognition.

[B27-behavsci-15-00134] Doebel S. (2020). Rethinking executive function and its development. Perspectives on Psychological Science.

[B28-behavsci-15-00134] Dong Y., Liu Y. (2016). Classes in translating and interpreting produce differential gains in switching and updating. Frontiers in Psychology.

[B29-behavsci-15-00134] Eakin L., Minde K., Hechtman L., Ochs E., Krane E., Bouffard R., Greenfield B., Looper K. (2004). The marital and family functioning of adults with ADHD and their spouses. Journal of Attention Disorders.

[B30-behavsci-15-00134] Friederici A. D. (2011). The brain basis of language processing: From structure to function. Physiological Reviews.

[B31-behavsci-15-00134] Friedman N. P., Miyake A. (2017). Unity and diversity of executive functions: Individual differences as a window on cognitive structure. Cortex.

[B32-behavsci-15-00134] Frischen U., Schwarzer G., Dege F. (2021). Music lessons enhance executive functions in 6-to 7-year-old children. Learning and Instruction.

[B33-behavsci-15-00134] Giguere D., Dickson D. J., Tulloch M. K., Hoff E. (2022). Majority language skill, not measures of bilingualism, predicts executive attention in bilingual children. Journal of Experimental Child Psychology.

[B34-behavsci-15-00134] Goodrich J. M., Koziol N. A., Yoon H., Leiva S. (2022). Do Spanish-english bilingual children outperform monolingual english-speaking children on executive function tasks in early childhood? A propensity score analysis. Journal of Educational Psychology.

[B35-behavsci-15-00134] Grady C. L., Luk G., Craik F. I. M., Bialystok E. (2015). Brain network activity in monolingual and bilingual older adults. Neuropsychologia.

[B36-behavsci-15-00134] Hackman D. A., Farah M. J. (2009). Socioeconomic status and the developing brain. Trends in Cognitive Sciences.

[B37-behavsci-15-00134] Han X., Li W., Filippi R. (2022). The effects of habitual code-switching in bilingual language production on cognitive control. Bilingualism-Language and Cognition.

[B38-behavsci-15-00134] Hannaway N., Opitz B., Sauseng P. (2019). Exploring the bilingual advantage: Manipulations of similarity and second language immersion in a Stroop task. Cognitive Neuroscience.

[B39-behavsci-15-00134] Hargreaves D. J., Aksentijevic A. (2011). Music, IQ, and the executive function. British Journal of Psychology.

[B40-behavsci-15-00134] Hayakawa S., Marian V. (2019). Consequences of multilingualism for neural architecture. Behavioral and Brain Functions.

[B41-behavsci-15-00134] Hilchey M. D., Klein R. M. (2011). Are there bilingual advantages on nonlinguistic interference tasks? Implications for the plasticity of executive control processes. Psychonomic Bulletin & Review.

[B42-behavsci-15-00134] Huang S., Seidman L. J., Rossi S., Ahveninen J. (2013). Distinct cortical networks activated by auditory attention and working memory load. NeuroImage.

[B43-behavsci-15-00134] Keijzer M. (2013). Working memory capacity, inhibitory control and the role of l2 proficiency in aging L1 dutch speakers of near-native L2 english. Brain Sciences.

[B44-behavsci-15-00134] Lehtonen M., Soveri A., Laine A., Jarvenpaa J., de Bruin A., Antfolk J. (2018). Is bilingualism associated with enhanced executive functioning in adults? A meta-analytic review. Psychological Bulletin.

[B45-behavsci-15-00134] Li P., Legault J., Litcofsky K. A. (2014). Neuroplasticity as a function of second language learning: Anatomical changes in the human brain. Cortex.

[B46-behavsci-15-00134] Lopez Zunini R. A., Morrison C., Kousaie S., Taler V. (2019). Task switching and bilingualism in young and older adults: A behavioral and electrophysiological investigation. Neuropsychologia.

[B47-behavsci-15-00134] Ma X., Ma X., Li P., Liu Y. (2020). Differences in working memory with emotional distraction between proficient and non-proficient bilinguals. Frontiers in Psychology.

[B48-behavsci-15-00134] MacDonald M. C., Christiansen M. H. (2002). Reassessing working memory: Comment on just and carpenter (1992) and waters and caplan (1996). Psychological Review.

[B49-behavsci-15-00134] Miyake A., Friedman N. P. (2012). The nature and organization of individual differences in executive functions: Four general conclusions. Current Directions in Psychological Science.

[B50-behavsci-15-00134] Miyake A., Friedman N. P., Emerson M. J., Witzki A. H., Howerter A., Wager T. D. (2000). The unity and diversity of executive functions and their contributions to complex “Frontal Lobe” tasks: A latent variable analysis. Cognitive Psychology.

[B51-behavsci-15-00134] Monnier C., Boiche J., Armandon P., Baudoin S., Bellocchi S. (2021). Is bilingualism associated with better working memory capacity? A meta-analysis. International Journal of Bilingual Education and Bilingualism.

[B52-behavsci-15-00134] Morton J. B., Harper S. N. (2007). What did Simon say? Revisiting the bilingual advantage. Developmental Science.

[B53-behavsci-15-00134] Nancy G., Bryson S. E., Smith I. M. (2008). Executive function in preschoolers: A review using an integrative framework. Psychological Bulletin.

[B54-behavsci-15-00134] Olulade O. A., Jamal N. I., Koo D. S., Perfetti C. A., LaSasso C., Eden G. F. (2016). Neuroanatomical evidence in support of the bilingual advantage theory. Cerebral Cortex.

[B55-behavsci-15-00134] Paap K. R., Johnson H. A., Sawi O. (2015). Bilingual advantages in executive functioning either do not exist or are restricted to very specific and undetermined circumstances. Cortex.

[B56-behavsci-15-00134] Privitera A. J., Momenian M., Weekes B. (2022). Task-specific bilingual effects in Mandarin-English speaking high school students in China. Current Research in Behavioral Sciences.

[B57-behavsci-15-00134] Privitera A. J., Momenian M., Weekes B. (2023). Graded bilingual effects on attentional network function in Chinese high school students. Bilingualism-Language and Cognition.

[B58-behavsci-15-00134] Ratiu I., Azuma T. (2014). Working memory capacity: Is there a bilingual advantage?. Journal of Cognitive Psychology.

[B59-behavsci-15-00134] Schwering S. C., MacDonald M. C. (2020). Verbal working memory as emergent from language comprehension and production. Frontiers in Human Neuroscience.

[B60-behavsci-15-00134] Snyder H. R. (2013). Major Depressive disorder is associated with broad impairments on neuropsychological measures of executive function: A meta-analysis and review. Psychological Bulletin.

[B61-behavsci-15-00134] Thorell L. B., Lindqvist S., Bergman Nutley S., Bohlin G., Klingberg T. (2009). Training and transfer effects of executive functions in preschool children. Developmental Science.

[B62-behavsci-15-00134] Xie Z., Pisano T. S. (2019). Second language (L2) proficiency, socioeconomic status (SES), and intelligence (IQ) are significant predictors of cognitive control differences among young adult unbalanced Chinese-English bilinguals. Bilingualism-Language and Cognition.

[B63-behavsci-15-00134] Xie Z., Zhou S. (2020). Bilingualism, demographics, and cognitive control: A within-group approach. Frontiers in Psychology.

[B64-behavsci-15-00134] Yang H., Hartanto A., Yang S. (2018). Bilingualism confers advantages in task switching: Evidence from the dimensional change card sort task. Bilingualism-Language and Cognition.

[B65-behavsci-15-00134] Zhou Y., Privitera A. J. (2024). Subjective versus objective language proficiency measures in the investigation of bilingual effects on cognitive control. International Journal of Bilingualism.

